# Immigrant family skills-building to prevent tobacco use in Latino youth: study protocol for a community-based participatory randomized controlled trial

**DOI:** 10.1186/1745-6215-13-242

**Published:** 2012-12-19

**Authors:** Michele L Allen, Diego Garcia-Huidobro, G Ali Hurtado, Rose Allen, Cynthia S Davey, Jean L Forster, Monica Hurtado, Katia Lopez-Petrovich, Mary Marczak, Ursula Reynoso, Laura Trebs, María Veronica Svetaz

**Affiliations:** 1 Department of Family Medicine and Community Health, School of Medicine, University of Minnesota, 717 Delaware Street S.E., Suite 166, Minneapolis, MN, 55414, USA; 2 Department of Family Social Science, University of Minnesota, 290 McNeal Hall, 1985 Buford Avenue, St Paul, MN, 55108, USA; 3 Center for Family Development, University of Minnesota Extension, 444 Coffey Hall, 1420 Eckles Avenue, St Paul, MN, 55108, USA; 4 Biostatistical Design and Analysis Center, Clinical and Translational Science Institute, University of Minnesota, 717 Delaware Street S.E., Minneapolis, MN, 55455, USA; 5 Division of Epidemiology and Community Health, School of Public Health, University of Minnesota, 1300 S. Second Street, Suite 300, Minneapolis, MN, 55454, USA; 6 Aqui Para Ti Clinic for Latino Youth, Hennepin County Medical Center, 2700 East Lake Street, Minneapolis, MN, 55406, USA; 7 Centro-El Zocalo, 1915 Chicago Avenue, Minneapolis, MN, 55404, USA

**Keywords:** Community-based participatory research, Hispanic Americans, Adolescent, Family, Smoking, Prevention and control

## Abstract

**Background:**

Despite declines over recent years, youth tobacco and other substance use rates remain high. Latino youth are at equal or increased risk for lifetime tobacco, alcohol, marijuana, and other illicit drug use compared with their white peers. Family plays an important and influential role in the lives of youth, and longitudinal research suggests that improving parenting skills may reduce youth substance use. However, few interventions are oriented towards immigrant Latino families, and none have been developed and evaluated using a community-based participatory research (CBPR) process that may increase the effectiveness and sustainability of such projects. Therefore, using CBPR principles, we developed a randomized clinical trial to assess the efficacy of a family-skills training intervention to prevent tobacco and other substance use intentions in Latino youth.

**Methods/Design:**

In collaboration with seven Latino community-serving agencies, we will recruit and randomize 336 immigrant families, into intervention or delayed treatment conditions. The primary outcome is youth intention to smoke 6 months post intervention. The intervention consists of eight parent and four youth sessions targeting parenting skills and parent–youth relational factors associated with lower smoking and other substance use in youth.

**Discussion:**

We present the study protocol for a family intervention using a CBPR randomized clinical trial to prevent smoking among Latino youth. The results of this trial will contribute to the limited information on effective and sustainable primary prevention programs for tobacco and other substance use directed at the growing US Latino communities.

**Trial registration:**

ClinicalTrials.gov: NCT01442753

## Background

The American Cancer Society has described cigarette smoking as the most preventable cause of premature death in the United States [1]. While smoking rates have declined among youth in the US over recent years, in 2005 one-half of all youth had tried cigarettes by the 12th grade [2]. Further, point prevalence data indicate that 8% of middle school students and 23% of high school students are current smokers [3]. Alcohol and illicit drug use have also decreased in recent years, but again remain at unacceptably high rates. That is, 75% of students have consumed more than a few sips of alcohol, and one-half have tried an illicit drug by the end of high school [2].

National data suggest that, compared with whites, middle-school-aged Latino adolescents are at equal or increased risk for lifetime tobacco, alcohol, marijuana, and other illicit drug use [2,4]. However, considering that the majority of Latino youth are first or second generation [5], and that rates of tobacco, alcohol, and other drug use increase in Latino youth with greater time in the US [6-8], rates of tobacco and other substance use will probably rise in future years without prevention efforts.

Protective effects against tobacco and other substance use seen in immigrant adolescents may be conferred by the norms, values, and behaviors that their parents bring from their countries of origin. For example, researchers have identified the traditional Latino value of familism – belief in the importance of family, including extended family [9] – as protective against alcohol use in youth [6,10]. Broader research on adolescent health suggests that parents have an important impact on their health behaviors [11], particularly among ethnic diverse groups [12,13]. Relationship characteristics, such as warmth, support, and acceptance, and parenting practices such as monitoring, discipline, and positive communication, protect against future tobacco, alcohol, and illicit drug use [11,14,15], and may be even stronger predictors of substance use for adolescents from diverse ethnicities [16]. However the quality of relationships and parenting practices are affected by immigration factors, which in turn affect youth substance use [17,18].

Because characteristics of the parent–youth relationship and parenting practices are associated with tobacco and other substance use outcomes, these family factors are common targets of parent training interventions [19]. The parent training interventions addressing these factors have shown a positive impact on predictor variables of tobacco and other substance use, such as increased parent involvement, child social competence, and child self-regulation, and on preventing and reducing adolescent tobacco, alcohol, marijuana and other substance use [20-22]. These interventions have the benefit of both enhancing protective factors and reducing risk [23,24].

While family-skills training programs have proven efficacious at preventing substance use, there continue to be challenges to widespread uptake of evidence-based programs [22]. One concern is that efficacy studies are often conducted in highly controlled conditions that may not attend to preferences or values of local communities, may not be feasible to replicate or sustain in community settings, and are delivered under conditions fundamentally different from practice environments [25]. Community-based participatory research (CBPR) approaches, which recognize the strengths, knowledge, expertise, and resources of communities, and so engage community members in the research process as full partners, may address these concerns by increasing the relevance, feasibility and cultural appropriateness of interventions [26-28]. Participatory approaches have also been suggested to improve the representativeness and to enhance the implementation of interventions by increasing the relevance and acceptability of intervention approaches and the methods and quality of delivery [29,30]. Although other family-skills training programs exist for Latino families, to our knowledge none have been developed using explicit CBPR principles in collaboration with organizations who would be probable end-users of the program [24,31].

Therefore, using a CBPR approach, we developed Padres Informados/Jóvenes Preparados (PI/JP) with the goal of preventing Latino youth substance use by improving parenting practices, parent–youth interpersonal skills, and youth social competencies. Throughout this paper we will refer to the heterogeneous population with heritage from South America, Central America, Mexico, and the Spanish-speaking Caribbean as Latino. Our community collaborative board recognizes that this label does not represent the diversity of ethnicities and ethnic identities held by people from these regions, but selected Latino as the most inclusive and commonly used term available. Here we describe the protocol to evaluate the program’s efficacy in preventing tobacco and other substance use intentions in middle-school-age Latino youth. Our outcome hypotheses are that, at 6 months post intervention, youth in the intervention arm will have statistically significant lower intention to use tobacco, alcohol, marijuana and other drugs, and their participant parents will have significantly better scores on parent–youth relationship and parenting practice measures compared with control adolescents and parents.

## Methods/Design

**Figure 1:**
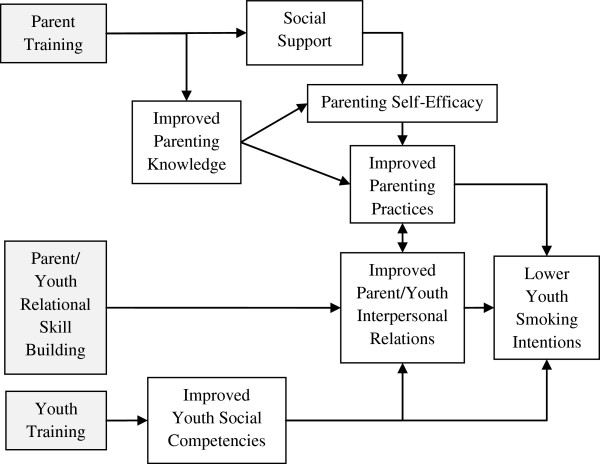
**Padres Informados/Jóvenes Preparados conceptual model.** *Intervention components in gray.

Based on social cognitive and human ecology theories, PI/JP was designed by a community collaborative board of university and community partners and a parent advisory board of Latino parents of youth (Figure 1). The process began with the parent advisory board, who identified core cultural values that informed their parenting practices and key parenting priorities. In addition, community collaborators directed decisions regarding feasibility of the intervention including limiting the program number of sessions, assuring accessible format and content, and, due to the locally limited number of bilingual/bicultural professionals, having the program delivered by professionals or experienced paraprofessionals. The community collaborative board spent 2 years developing the eight-session curriculum integrating parent and community priorities and practical family education strategies with theoretical behavior change models. Later, a pilot study was successfully conducted to evaluate feasibility of the training program and to make final curriculum changes [32].

The community collaborative board was expanded to include seven participating community agencies: five urban and two rural agencies, including two clinics, a school, three social service agencies and a University of Minnesota Extension site. Agencies serve a largely Latino constituency and hold reputations as trusted service providers. All agencies agreed to participate in the planning, implementation, and evaluation of the project as is consistent with CBPR principles [33].

The study will proceed through a planning and training phase (year 1), a serial implementation phase (years 2 to 4), and a data analysis and dissemination phase (year 5). To increase community capacity for supporting family-skills training and participating in future research collaborations, collaborating agencies are responsible for recruitment of participants and delivery of the intervention. Therefore, each collaborative site will select two staff to directly participate in the project: one to recruit and coordinate program activities, and a second to deliver the intervention. Community collaborators will be prepared for their roles through extensive training in protection of research participants’ confidentiality, the content of the program, and the theoretical background and practical skills needed for intervention implementation. Criteria for site-facilitators who will deliver the intervention include: preference for degree in education, psychology/therapy, or social work, but left to the discretion of site; at least 3 years of experience providing direct service to Latino families; and bicultural and bilingual in English/Spanish.

### Design overview

**Figure 2:**
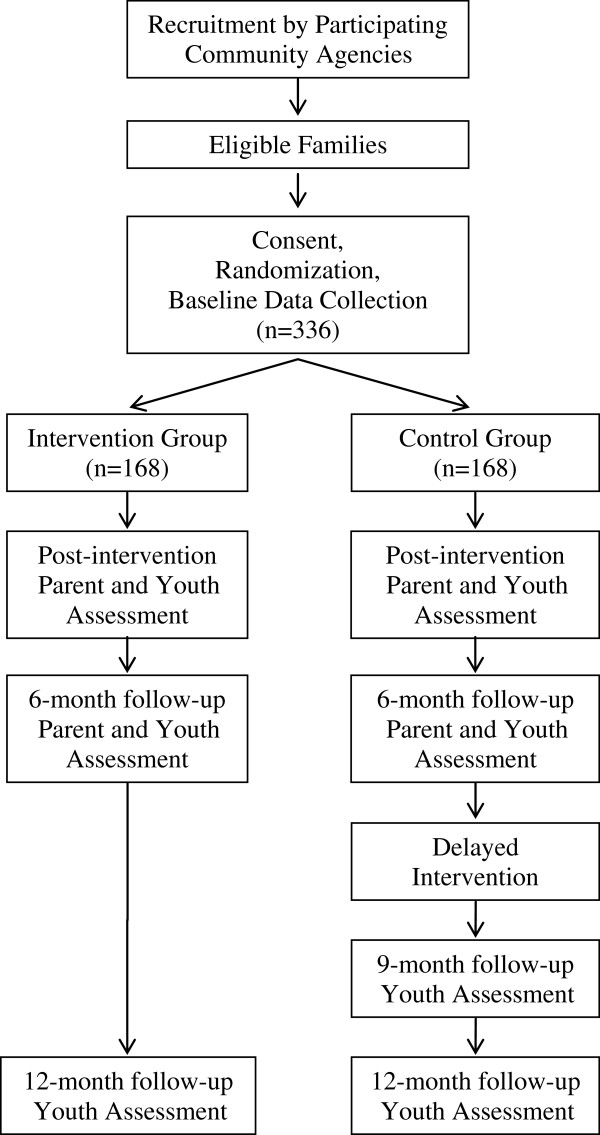
Padres Informados/Jóvenes Preparados study design.

We will conduct a randomized controlled trial (RCT) with a delayed treatment condition for the control group (Figure 2) to evaluate the efficacy of the PI/JP family skills training program at preventing tobacco and other substance use intentions in Latino youth. As has been true in other participatory studies, a delayed control trial was determined by community partners to be more acceptable than a traditional RCT [34].

Participants will be enrolled from the seven collaborating agencies. Recruitment will be facilitated by the community agencies with support from the university research team. Family eligibility criteria are listed in Table 1. Randomization will occur at the level of the family with a goal of enrolling 336 families (one youth aged 10 to 14 and at least one parent/guardian) into the study. Families assigned to the intervention condition will receive the family-skills training immediately, while those in the control condition will receive the training approximately 6 months after the first group. To limit the possibility of contamination, facilitators are directed to not utilize materials or procedures outside the study, and parents are asked to not discuss study content with anyone other than a co-parent.

**Table 1:** Eligibility criteria for participants

**Inclusion criteria**	
Parent	
	Either mother or father born in a Latin American country
	Speaks Spanish
	Willing to give consent for self and youth
Youth	
	Between ages 10 and 14
	Speaks English or Spanish
	Identifies himself/herself as Latino/Latina
	Willing to give assent
**Exclusion criteria**	
	Mental disorder incompatible with the participation on the trial
	Prior participation in Padres Informados/Jóvenes Preparados pilot or planning

The primary outcome is intention to use cigarettes in the participating youth, which will be operationalized as susceptibility to smoking assessed among never-smokers and puffers from their responses to three questions (never-smokers) or two questions (puffers) [35,36]. These questions are: ‘Do you think you will try a cigarette soon?’ (not asked of puffers), ‘Do you think you will be smoking cigarettes one year from now?’, and ‘If one of your best friends were to offer you a cigarette, would you smoke it?’ A never-smoker will be considered nonsusceptible if the answer to the first question is ‘no’ and answers to the both the second and third questions are ‘definitely not’; a puffer will be considered nonsusceptible if answers to the second and third questions are ‘definitely not’. Additional outcomes of alcohol, marijuana and other substance intentions, and past 30-day use of all substances will be assessed through items previously used with middle-school-aged Latino youth [37,38]. Secondary outcomes include parenting practice and parent–youth interpersonal relational measures that are known to be important predictors of substance use among youth. Table 2 presents the instruments used to evaluate each outcome. We chose scales with established reliability and validity, availability in Spanish, and that were appropriate for low-literacy groups. Most scales include parallel items to be completed by both parents and youth. Bilingual data collectors will lead group interviews using paper-and-pencil surveys with adults and self-administered computer-assisted survey interviewing with youth.

**Table 2:** Instruments used to measure parenting practices and parent–youth relationships

**Scale**	**Cronbach’s alpha in Mexican/Latino population (reference)**
Parenting practices	
Parental Monitoring Knowledge	0.78 to 0.82 [39-41]
Parental Acceptance	0.67 to 0.79 [42]
Consistent Discipline	0.69 to 0.77 [43]
Parenting Practices Self-efficacy	0.86 to 0.94 [44]
Multidimensional Scale of Perceived Social Support	0.93 to 0.86 [45-49]
Parent–youth relationships	
Parent**–**Youth Positive Attachment	0.81 to 0.85 [50]
Adolescent Parent–Child Conflict/Conflict Processes	0.87 to 0.92 [51]

Incentives for participation for parents and youth will be tied to data collection points. Parents will be offered $30 and youth $25 dollar gift cards upon completion of each survey, independent of their participation in the intervention.

This protocol and the corresponding consent forms were approved by the Institutional Review Board of the University of Minnesota (HS# 1008S86938) and the Hennepin County Medical Center Institutional Review Board (HSR# 11–3409).

### Intervention

The PI/JP curriculum aims to develop strong parenting practices and facilitate relationship-building between parents and youth, while emphasizing Latino cultural values, navigation in multiple cultures, and environmental risks related to socioeconomic circumstances. As outlined in Table 3, the curriculum is comprised of eight 3-hour sessions including 30 minutes for dinner and conversation to facilitate social support among parents. Four sessions include parents only, one session includes parents and youth in independent topics, and three sessions include parents and youth in parallel topics with joint skill-building sessions at the end. All sessions are learner focused and follow an experiential learning approach, integrating participants’ reflections, didactics, skill-building activities, and group discussions.

**Table 3:** Description of the intervention sessions

**Session**	**Participants**	**Goals for the session**	**Example activities**
1 Parenting Styles	Parents	Parents will:	Parents will:
		· Recognize their own parenting style.	· Review a video on parenting styles and discuss.
		· Distinguish the key elements of the positive parenting style.	· Self-assess their parenting styles and discuss in small groups.
2 Between Multiple Worlds	Parents	Parents will:	Parents will:
		· Recognize that their strong family orientation as Latino parents protects against youth risk behavior.	· Identify their own cultural values as parents and compare/contrast with those of school and youth.
		· Understand that their youth must navigate between family, school, and peer cultures.	· Self-assess their own and their youth’s cultural orientation and reflect on differences.
		· Learn skills to help their youth navigate across cultures.	
3 Adolescent Development	Parents	Parents will:	Parents will:
		· Review normal adolescent development and the need to adapt parenting strategies to developmental stages.	· Compare the behaviors of their youth with previous years.
		· Contextualize challenging behavior within tasks of adolescent development.	· Reflect on why adolescents challenge parents and how not to ‘take it personally’.
4 Communication	Parents and youth	Parents will:	Parents will:
		· Understand how strong communication fits with the values of respect for authority and *confianza* (trusting relationships).	· Practice active listening.
			· Discuss barriers and facilitators to conversations with youth.
		· Learn basic principles of good communication and develop communication skills.	· Practice communication using ‘I’ messages.
		Youth will:	Youth will:
		· Recognize the importance of communication with parents.	· Play the broken phone and identify barriers in communication.
		· Learn specific communication skills.	· Practice when and how to talk and listen.
			· Practice ‘I’ messages.
5 Discipline	Parents	Parents will:	Parents will:
		· Learn the importance of positive behavioral reinforcement.	· Identify strategies to encourage positive behavior in their youth.
		· Understand the importance of establishing negotiable and non-negotiable rules, and establishing and reinforcing consequences.	· Practice developing negotiable and non-negotiable rules for their family.
6 Conflict resolution	Parents and youth	Parents will:	Parents will:
		· Understand that conflict is part of the normal developmental process, and does not need to damage their relationships.	· Identify positive and negative aspects of conflicts.
		· Identify collaborative problem-solving and conflict resolution strategies.	· Discuss and role-play a strategy to solve problems with their youth.
		· Learn to recognize and self-regulate emotions.	· Develop strategies to manage anger.
		Youth will:	Youth will:
		· Learn collaborative problem-solving strategies.	· Talk about rules, consequences and mistakes.
		· Identify strategies to recognize and manage emotions.	· Talk about emotions and their consequences.
			· Name the steps to effectively solve problems.
7 Supervision and Friends’ Influence	Parents and youth	Parents will:	Parents will:
		· Understand the meaning and importance of monitoring.	· Discuss scenarios to differentiate supervision from over-control.
		· Learn strategies to monitor their youth effectively at each developmental stage of adolescence.	· Role-play initiating conversations with parents of their children’s friends.
		Youth will:	Youth will:
		· Identify dreams that they want to achieve.	· Develop a collage to help visualize dreams and goals.
		· Identify influences and behavior (including substance use) that may get in the way of achieving dreams.	· Practice strategies to refuse risky behaviors.
		· Learn strategies to avoid problem behaviors.	
8 Connection	Parents and youth	Parents will:	Parents will:
		· Understand that parent–youth connection is the foundation for parenting, and recognize barriers to relationship-building.	· Engage in activity around setting kids as priority for time.
		· Identify strategies to strengthen their relationship with their youth.	· Write a letter to their children expressing their love and expectations.
		Youth will:	Youth will:
		· Reflect on the importance of a strong parent–youth relation.	· Complete a map of personal connections.
		· Identify parents and other adults as a support network.	· Write a message to their parents expressing their thankfulness.

Each parent session will begin with identification of a challenge the parent has experienced in the last week with their youth related to the topic of the day. The intent of this exercise is to ground the didactic and skill-building materials in a problem relevant to the family’s current situation. Facilitators will then ask parents to reflect on their own topic-specific experiences as a youth to generate empathy in the parents for their children’s situations and challenges. The remainder of the session will focus on a combination of presentation of didactic information, sharing, and skill-building exercises. The skill-building exercises largely use tobacco and other substance use as examples, thus infusing all sessions with practical means for preventing substance use. The sessions end with parents reflecting on how the information and skills they learned may be applied to the challenge with their youth they identified at the beginning of the session. Parents then set an achievable goal for the week to address this challenge, and at the beginning of the following session share their experiences implementing this goal.

Youth sessions begin with an icebreaker activity and a debriefing period that introduces the day’s topic with the dual intent of cultivating a youth-friendly atmosphere and also metaphorically relating the topic of the day to a concrete idea. Introduction of new concepts follows, intermingled with a variety of theme-related activities, skill-building exercises and role-playing, helping to solidify the proposed topic of the day. The youth curriculum was formatted to rotate between didactic moments, key conversations, and activities in order to be continuously engaging and appropriately meeting the youth’s needs as adolescent learners.

Sessions using the parallel parent–youth format are communication, conflict resolution, and connection. Involving youth in the relationship-oriented sessions is intended to invest both parents and youth in building solid relationships that facilitate successful parenting practices [19]. For these sessions, parents and youth initially meet independently. The two groups will come together at the end of the sessions for joint skill-building exercises and discussion. For the parent–youth session without joint activities, parents will focus on parental monitoring while youth will build basic youth social competencies such as goal-setting, and refusal skills that have been associated with lower tobacco and other substance use behaviors [52,53].

Because the proposed program was developed in collaboration with community agencies, the likelihood of close adherence to the intervention protocol is high. However, fidelity will be assured through extensive training, observation of training, and delivery of structured feedback for facilitators delivering the intervention. A detailed checklist addressing implementation factors will be used to evaluate fidelity, including appropriate delivery of key content for each session, and utilization of study materials. All sessions will be videotaped and a random selection of 40% of the session tapes representative of all sites will be reviewed using the checklist. Summary fidelity scores will be generated for each implementation factor.

### Statistical analyses

#### Sample size and power

Baseline susceptibility rates of 22% for both groups and a 6-month absolute increase of 6% in the control group are consistent with susceptibility rates and likelihood of tobacco use rates observed in other studies for youth in this age range [24,36,54]. We will anticipate a post-intervention absolute decrease of 14% in the likelihood of youth tobacco use [24]. Using a conservative estimate of an 8% decrease in smoking intention in the intervention group, compared with a 6% increase in the control group at 6 months, a sample size of 140 per group provides 80% power to detect this difference as significant using a two-tailed chi-square test of proportions with significance level (alpha) of 0.05. Anticipating a loss to follow-up of 18%, we will initially enroll and randomize 336 families. A difference ≥0.20 in mean pre/post changes in parenting behavior scores represents a difference comparable with those found in evidence-based programs listed on SAMHSA’S national registry, [55,56] and is consistent with differences observed in the feasibility study.

#### Data analysis

The primary outcome, susceptibility to smoke cigarettes, will be evaluated by a multiple logistic regression model to generate an adjusted odds ratio testing the null hypothesis that the 6-month post-intervention susceptibility rate is the same in both groups. The 6-month post-intervention susceptibility to use other substances also will be compared between intervention and control groups.

Mixed-effect models utilizing repeated measures of smoking susceptibility, and including intervention as a time-varying covariate, will be used to evaluate intervention effects on growth trajectories of susceptibility to smoking. Both linear and nonlinear models will be explored to identify the most appropriate characterizations of changes over time in susceptibility to smoking for each group.

The secondary outcomes, self-reported parenting practices and parent–youth interpersonal relations, will be evaluated by analysis of covariance models. In addition, within-group changes over time in mean parenting and relationship scale scores will be assessed using repeated-measures analysis of variance.

#### Missing data strategies for primary and secondary outcomes

Only youth with 6-month post-intervention susceptibility responses will be included in the logistic regression analysis of the primary outcome as there is no reliable method to impute data for this outcome. Multiple imputations will be used for secondary outcomes for subjects with ≤45% of items missing on a given scale. Comparisons of baseline characteristics between study completers and those lost to follow-up will be performed using chi-square tests and two-sample *t*-tests. In addition, sensitivity analyses of the primary outcome will be performed to assess the robustness of intervention effect estimates under a variety of possible attrition bias scenarios [57].

Finally, according to CBPR we will involve the community partners in the result interpretation process. Together we will formulate a dissemination plan to return results to other interested community agencies, study participants, and the larger community.

## Discussion

There is an important need to develop culturally sensitive interventions to prevent smoking initiation, especially among Latino youth. Because familism is an important value for the Latino community [9], and family relational characteristics such as warmth, support, acceptance, positive parenting practices, and positive communication are stronger predictors of substance use behaviors in Latino adolescents [16], including the family is fundamental for effective preventive interventions in this group. Therefore, this study is directed to promote positive parent–youth relationships and effective parenting practices to finally impact youth smoking, alcohol, and drug-use initiation.

To tailor the program to the specific needs of the Latino community, and to enhance dissemination of the program in community agencies, we utilized a CBPR approach. To our knowledge, this is the first family-skills training program in which community members are active participants in the research project and where CBPR principles have been explicitly acknowledged. Therefore, this RCT will provide insights not only for the efficacy of the designed intervention, but also for the collaborative factors that determine successful implementation. This information is essential for implementation and dissemination of effective interventions [28], particularly in underserved communities.

This study also has limitations. Because participants will be enrolled from organizations in a single Midwestern state, results may not generalize to Latino families in other regions of the US. However, research on Latino populations in areas of recent settlement is rare, so this information may contribute new knowledge regarding how to prevent substance use in these rapidly growing and under-researched populations.

While many potential benefits are likely to result from a CBPR approach to developing and implementing the intervention, there are also a number of potential limitations related to this design. One concern is contamination, given that families being served by these organizations may know each other. Because the time investment in the parenting classes is significant, classes build on each other, and the program includes self-reflection, discussion and skill-building activities, it is unlikely that parents casually receiving knowledge from intervention parents would experience substantive change in skills. Additionally, because community partners requested having a control group with a delayed intervention, we will not be able to assess long-term effects of the intervention as in a traditional RCT [34]. Additionally, use of nonresearch staff from a range of experiential and professional levels to deliver the intervention may lead to lower fidelity to key intervention components. We will ameliorate this risk by providing extensive training and support to site facilitators. The potential benefits of improved knowledge regarding implementation in community settings and increased community capacity-building outweigh these risks.

We present the study protocol of a family intervention using a CBPR randomized clinical trial to prevent smoking among Latino youth. The results of this trial will contribute to the limited information on effective smoking primary prevention programs directed to the growing Latino community in the US and will provide insights into effective strategies to reduce health disparities in this underserved population.

## Trial status

The trial is currently implementing the control group intervention (two agencies) and performing follow–up data collection (two agencies). Four agencies will initiate recruitment during early 2013.

## Abbreviations

CBPR: community-based participatory research; PI/JP: Padres Informados/Jóvenes Preparados; RCT: randomized controlled trial.

## Competing interests

The authors declare that they have no competing interests.

## Authors’ contributions

MLA, JLF, CSD, MH, MM, KL-P, LT, RA, and MVS participated in the design of the study. CSD led the statistical and power calculations. MLA, DG-H, GAH, KL-P, RA, LT, UR, and MVS are participating in the development and implementation of the intervention. GAH is coordinating the study. MLA, DG-H and GAH led the redaction of this article. All authors read, revised and approved the final manuscript.
